# IDO Downregulation Induces Sensitivity to Pemetrexed, Gemcitabine, FK866, and Methoxyamine in Human Cancer Cells

**DOI:** 10.1371/journal.pone.0143435

**Published:** 2015-11-18

**Authors:** Saman Maleki Vareki, Di Chen, Christine Di Cresce, Peter J. Ferguson, Rene Figueredo, Macarena Pampillo, Mateusz Rytelewski, Mark Vincent, Weiping Min, Xiufen Zheng, James Koropatnick

**Affiliations:** 1 Department of Microbiology and Immunology, Western University, London, Ontario, Canada; 2 Department of Pathology, Western University, London, Ontario, Canada; 3 Department of Oncology, Western University, London, Ontario, Canada; 4 Department of Physiology and Pharmacology, Western University, London, Ontario, Canada; 5 Cancer Research Laboratory Program, Lawson Health Research Institute, London, Ontario, Canada; Wayne State University School of Medicine, UNITED STATES

## Abstract

Indoleamine 2,3-dioxygenase-1 (IDO) is an immune regulatory enzyme expressed by most human tumors. IDO levels in tumor cells correlate with increased metastasis and poor patient outcome and IDO is linked to tumor cell resistance to immunotherapy, radiation therapy, and chemotherapy. Knowledge of tumor cell-autonomous effects of IDO, independent of its well-known role in regulating and suppressing anti-tumor immune responses, is limited. Clonal populations of A549 human lung adenocarcinoma cells stably transfected with anti-IDO shRNA or scrambled control shRNA were used to study IDO effects on drug sensitivity and resistance. IFNγ was used to induce IDO in those cells. We show, for the first time, that IDO mediates human tumor cell resistance to the candidate anticancer drugs FK866 (an NAD^+^ inhibitor), methoxyamine (MX, a base excision repair [BER] inhibitor) and approved anticancer drugs pemetrexed (a folate anti-metabolite) and gemcitabine (a nucleoside analogue), and combined treatment with pemetrexed and MX, in the absence of immune cells. Concurrent knockdown of IDO and thymidylate synthase (TS, a key rate-limiting enzyme in DNA synthesis and repair) sensitizes human lung cancer cells to pemetrexed and 5FUdR to a greater degree than knockdown of either target alone. We conclude that BER in IDO-expressing A549 cells plays a major role in mediating resistance to a range of approved and candidate anticancer drugs. IDO inhibitors are undergoing clinical trials primarily to improve antitumor immune responses. We show that targeting IDO alone or in combination with TS is a potentially valuable therapeutic strategy for cancer treatment, independent of immune activity and in combination with conventional chemotherapy.

## Introduction

The immunoregulatory molecule IDO is a 45 kDa hemoprotein essential for oxidative catabolism of tryptophan in the kynurenine pathway [1]. IDO catalyzes oxidative cleavage of the 2,3-double bond in the indole moiety of L-tryptophan, resulting in the production of the first kynurenine pathway metabolite, N-formyl kynurenine [2]. The final product of the kynurenine pathway is quinolinic acid (QA) that can be converted to NAD^+^ in mammalian cells. We and others have shown that IDO provides a source of NAD^+^ to cells from tryptophan catabolism [3,4]. IDO can be induced in most human cells, especially antigen-presenting cells (APCs), by inflammatory cytokines such as interferon gamma (IFNγ), tumor necrosis factor (TNF)-α, and infection [5,6]. However, most human tumors express IDO [7], which contributes to tumor-induced tolerance and suppression of the immune system. IDO induces a tolerogenic state in the tumor microenvironment and tumor-draining lymph nodes [8].

In the majority of patient studies, IDO expression has been correlated with decreased overall survival and decreased progression-free survival [9]. Moreover, IDO has been linked to increased metastasis in various human cancers including non-small cell lung carcinoma (NSCLC), breast cancer, and colorectal cancer [10–12]. Additionally, patients with advanced stage ovarian cancer, nasopharyngeal carcinoma, and endometrial cancer had high IDO levels in their tumors [13].

IDO is also important in developing resistance to immunotherapy. It has been suggested that IDO plays a major role in resistance to ipilimumab [14]. In a mouse transgenic model of breast cancer in which tumors were induced by expression of the oncogene Neu under the control of the mouse mammary tumor virus (MMTV) promoter, IDO inhibition with 1-methyl tryptophan (1-MT) was combined with paclitaxel, a chemotherapeutic agent commonly used to treat breast cancer [15]. The combination resulted in tumor regression in tumor-bearing animals [15]. Strikingly, depletion of CD4^+^ T cells or the use of T cell-deficient athymic mice instead of immunocompetent mice abolished the effect of combined treatment, indicating an immune-mediated effect for blocking IDO in the context of paclitaxel treatment [15].

Several clinical studies have suggested that high IDO levels during treatment could be related to poor outcome to chemotherapy and/or radiotherapy and, perhaps, contribute to resistance to therapy [16–18]. In a single arm Phase II study in patients with stage III NSCLC, patients were treated with induction gemcitabine followed by concurrent carboplatin, paclitaxel, and 74 Gray (Gy) thoracic radiation [16]. Cancer patients showed high IDO activity as implied by measured higher serum kynurenine/tryptophan ratios compared to healthy controls. This high IDO activity after chemotherapy was associated with poor patient outcome, although the statistical power of the study was limited by the relatively low number of patients [16].

In another study, IDO was positively associated with chemoresistance in a gene expression profiling study aimed at identifying molecules associated with resistance to paclitaxel-based chemotherapy in ovarian cancer cell lines and refractory surgical ovarian cancer specimens [17]. IDO was highly expressed in both paclitaxel-resistant cell lines and refractory ovarian tumors but was absent in paclitaxel-sensitive cell lines and tumors [17].

In a clinical study that analyzed NSCLC patient response to platinum-based chemotherapy in a small cohort of patients, IDO expression in monocytes and granulocytes was analyzed pre- and post-treatment. The patient population that benefited from the treatment showed lower IDO expression in blood monocytes post-treatment [18].

We have shown that IDO confers resistance to cisplatin, olaparib, and γ-radiation in A549, Hela, and H441 cells, independent of direct immune involvement [4]. IDO downregulation also decreased intracellular NAD^+^ levels in cancer cells [4]. NAD^+^ is necessary for poly(ADP-ribose) polymerase (PARP) function [19]. Recruitment of the BER scaffold protein X-ray repair cross-complementing protein 1 (XRCC1) to the damaged area of DNA is strictly dependent on poly-ADP-ribosylation [20]. PARP function therefore directly impacts BER, a critical mediator of cancer cell resistance to the genotoxic effects of γ-radiation and a number of chemotherapy agents including cisplatin, pemetrexed, and gemcitabine [21,22]. Therefore, IDO expression in cancer cells could potentially enhance BER in these cells and induce resistance to such agents.

We show, for the first time, that IDO expression in cancer cells, independent of the immune system, confers resistance to the NAD^+^ inhibitor FK866 and the BER inhibitor MX. Moreover, IDO downregulation sensitized cancer cells to pemetrexed and gemcitabine, both of which target thymidylate synthase (TS), but not the TS-targeting drug 5FUdR. IDO downregulation also sensitized A549 cells to combined pemetrexed and MX treatment. Finally, simultaneous downregulation of IDO and TS enhanced the capacity of IDO downregulation and TS downregulation to sensitize A549 cells to pemetrexed and 5FUdR, respectively.

## Materials and Methods

### Cell Culture

Human lung adenocarcinoma A549 cells were obtained from the American Type Culture Collection (ATCC, CCL-185), and maintained in minimal essential medium α (MEMα). A549 cells were STR fingerprinted and validated by Radil test to be mycoplasma free. Cultured media were supplemented with 10% fetal bovine serum (FBS)(Gibco, Life Technologies, Carlsbad, California, USA, catalogue # 325-043-EL), 100 units/ml penicillin and 100 μg streptomycin (pen/strep)(Gibco, Life Technologies, Carlsbad, California, USA, catalogue # 15140–122) in 70 cm^2^ flasks Sigma-Aldrich (St. Louis, Missouri, USA). Cells were maintained at 37°C in 5% CO2. For most experiments, cells were allowed to proliferate to no more than 70–80% of maximum occupancy on tissue culture plastic (*i*.*e*., 70–80% confluent).

### Proliferation Assay (Cell Counting)

Tumor cell proliferation after siRNA transfection and/or drug treatment was described previously [23]. Briefly, A549 cells were washed with PBS, trypsinized, and counted using a Beckman Coulter Z1 Particle Counter (Beckman, Mississauga, Ontario) 72 h after treatment. The fold change in cell number after 3 days of growth (relative to the starting number of plated cells) was calculated as:

$$\text{Fold Change= }\frac{\left[\left(\text{Number of Cells, Day 3}\right)-\left(\text{Number of Cells, Day 0}\right)\right]}{\left(\text{Number of Cells, Day 0}\right)}$$

Differences in proliferation induced by treatment were expressed as “Proliferation (% control)” and were calculated according to the following formula:

$$\text{Proliferation (\% Control) = }\left(\frac{\text{Treatment Fold Change}}{\text{Control Fold Change}}\right)\times 100$$

### Cytotoxic Drugs

5FUdR was purchased from Sigma Chemical Co. (St. Louis, Missouri, USA). Pemetrexed (Alimta, Eli Lilly and Co., Toronto, Ontario, Canada) was obtained from the London Health Sciences Centre pharmacy (London, Ontario, Canada). MX and FK866 were purchased from Sigma-Aldrich (St. Louis, Missouri, USA).

### TS Protein Detection and Measurement

A549 cells were cultured in 75 cm^2^ flasks. Cells were incubated for 96 h post TS siRNA transfection, washed twice with ice-cold PBS, harvested, and sonicated. Lysed cells were centrifuged at 20000 X g for 15 min at 4°C and the supernatant collected and stored at -80°C for future use. Protein extracts (20 μg) were quantified by BioRad protein assay, separated by electrophoresis through a 12% polyacrylamide gel, and then electro-transferred to a nitrocellulose membrane. TS monoclonal antibody (Taiho Pharmaceutical, Hanno-City, Japan) was kindly provided by Dr. Masakazu Fukushima (Taiho pharmaceuticals, Hanno Research Center, Hanno-City, Japan). Actin monoclonal antibody (Sigma, St. Louis, MO) was used to detect and quantify actin. Secondary anti-mouse and anti-rabbit IgG (peroxidase-linked whole antibodies; GE Healthcare Life Sciences) were bound to primary IDO and actin antibodies, respectively. The antibody-protein complexes were visualized using a Storm scanner (GE Healthcare Life Sciences).

### Pemetrexed, FK866, Methoxyamine, and 5FUdR Treatment

A549 cells (5x10^4^) were seeded into 25 cm^2^ flasks in 2 ml of MEMα supplemented with 10% FBS plus pen/strep. Medium was replaced with fresh growth medium with or without IFNγ (25 ng/ml) 16–24 h after seeding. Twenty-four or 48 h after addition of IFNγ, medium was replaced with fresh medium containing either pemetrexed (200 nM), FK866 (5 nM), MX (3 mM) or 5FUdR (40 nM). Three days after addition of drugs, cells were washed to remove the dead cells and particles. Adherent cells were trypsinized and enumerated using a Coulter counter (Beckman, Mississauga, ON). Viability of the counted cells was confirmed by trypan blue exclusion.

### Combined Treatment with Pemetrexed and Methoxyamine

A549 cells (5x10^4^) were grown and co-treated with pemetrexed (30 nM) and MX (3 mM) as described above. Cells were allowed to proliferate in culture for 72 h. Cells were then trypsinized and live cells were enumerated using a Coulter counter.

### IDO downregulation

Human A549 cells were stably transfected with short hairpin RNA (shRNA) with a scrambled control sequence non-complementary to known human RNA sequences, or antisense to human IDO1 or (SuperArray, Mississauga, ON; using Lipofectamine 2000 (LFA2K)(Invitrogen, Burlington, ON, Canada), and individual clonal populations with scrambled control (scr) shRNA or anti-IDO shRNA isolated as described previously [4]. Briefly, one million A549 cells were seeded overnight in 2 ml of MEMα supplemented with 10% FBS. Cells were 70% confluent on transfection day. Ten micrograms of anti-IDO shRNA plasmid or the scrambled control shRNA plasmid were mixed with 10 μl LFA2K and 125 μl serum-free medium. After 20 min of incubation at room temperature the shRNA: LFA2K complex was added to the cells. Transfection continued for 4 h before adding 4 ml of fresh MEMα supplemented with 10% FBS. Twenty-four h after transfection, cells were transferred to 14 cm plastic tissue culture dishes containing 20 μg puromycin (Bioshop Canada, Inc., Burlington, ON). Single colonies were selected and grown in 48-well plates in the presence of 2 μg puromycin. IDO mRNA and protein were measured in all selected clones after induction with IFNγ.

### TS and BRCA2 siRNA Transfection and Drug Treatment

TS siRNA number 3 or TS siRNA number 4 (Table 1), targeting different regions of human TS mRNA [OnTarget Plus (Dharmacon RNAi Technologies, Lafayette, CO, USA)] and that reduced target mRNAs by approximately 70% by 24 h after transfection, were used to transiently downregulate TS mRNA in A549 cancer cells. Concentrations of siRNAs targeting human breast cancer type-2 susceptibility protein (BRCA2) [OnTarget Plus SMARTPool BRCA2 (Dharmacon RNAi Technologies)](Table 1) that transiently reduced target mRNAs by approximately 70% by 24 h after transfection were determined (10 nM). TS siRNA (5 nM), BRCA2 siRNA (10 nM), and control non-targeting siRNA (2.5 or 5 nM) in serum-free MEMα and LFA2K (2.5 μg/ml) were incubated together for 20 min. The siRNA:LFA2K mix was then added to A549 cells that had been seeded, in triplicate, at 2 x10^5^ cells per 25 cm^2^ flask 24 h beforehand. At 4 h after addition of siRNA:LFA2K, media was exchanged for fresh growth medium containing IFNγ (25 ng/ml). Medium was replaced 48 h later with fresh medium containing pemetrexed or 5FUdR. Tumor cell proliferation was enumerated 72 h later using an an electronic particle counter (Beckman-Coulter).

**Table 1:** 1. ON-Target Plus^®^ TS and BRCA2 siRNA target mRNA sequences.

siRNA ID	Targeted RNA	Target mRNA Sequence	Target Position in mRNA Transcript
TS #3	TS mRNA	5′-ACAGAGAUAUGGAAUCAGA-3′	576–594
TS #4	TS mRNA	5′-GGACUUGGGCCCAGUUUAU-3′	526–544
BRCA2	BRCA2 mRNA	5′-GAAACGGACUUGCUAUUUA-3′	4285–4303
BRCA2	BRCA2 mRNA	5′-GGUAUCAGAUGCUUCAUUA-3′	558–576
BRCA2	BRCA2 mRNA	5′-GAAGAAUGCAGGUUUAAUA-3′	1949–1967
BRCA2	BRCA2 mRNA	5′-UAAGGAACGUCAAGAGAUA-3′	7242–7260
Control	No Target	5’-UGGUUUACAUGUUGUGUGA-3’	Not applicable

### *In vivo* Tumor Model

A549 clones NC-3 and 2–18 were described previously [4]. Tumor cells were injected in 50 per cent Matrigel (ECM gel, Sigma-Aldrich, St. Louis, MO) into flanks of Fox Chase SCID mice (1 x 10^7^ cells per injection site)(n = 5 mice per group). Once tumors reached ~300 mm^3^ mice were treated (day 0) with PBS-diluted recombinant human IFNγ (100,000 IU, i.p., two times per week for four weeks)(R&D Systems, 285-IF/CF, Minneapolis, MN) and/or PBS-diluted pemetrexed (50 mg/kg, i.p., once per week for four weeks). Tumor volumes were estimated from caliper measurements of length and width and calculated according to the following formula: π/6 x (longest diameter) x (shortest diameter)^2^. Animal handling and procedures were conducted according to the animal experimentation guide and protocols of the Animal Use Subcommittee of the University of Western Ontario.

### Statistical Analysis

Student’s *t* test (2-tailed) was used to determine differences between two means. One-way ANOVA was used to assess differences among multiple means. A *p* value of 0.05 was selected *a priori* to indicate significant differences. In some analyses, data were pooled from A549 clonal populations that expressed anti-IDO shRNA and compared to the pooled measurements of multiple clones expressing scrambled control shRNA. In certain cases, the strength of linear correlation between IDO protein and cell proliferation was determined by calculating the coefficient of determination (r^2^) between the 2 variables using Graphpad Prism 6.

## Results

### IDO confers resistance to NAD^+^ inhibitor FK866

**Fig 1:**
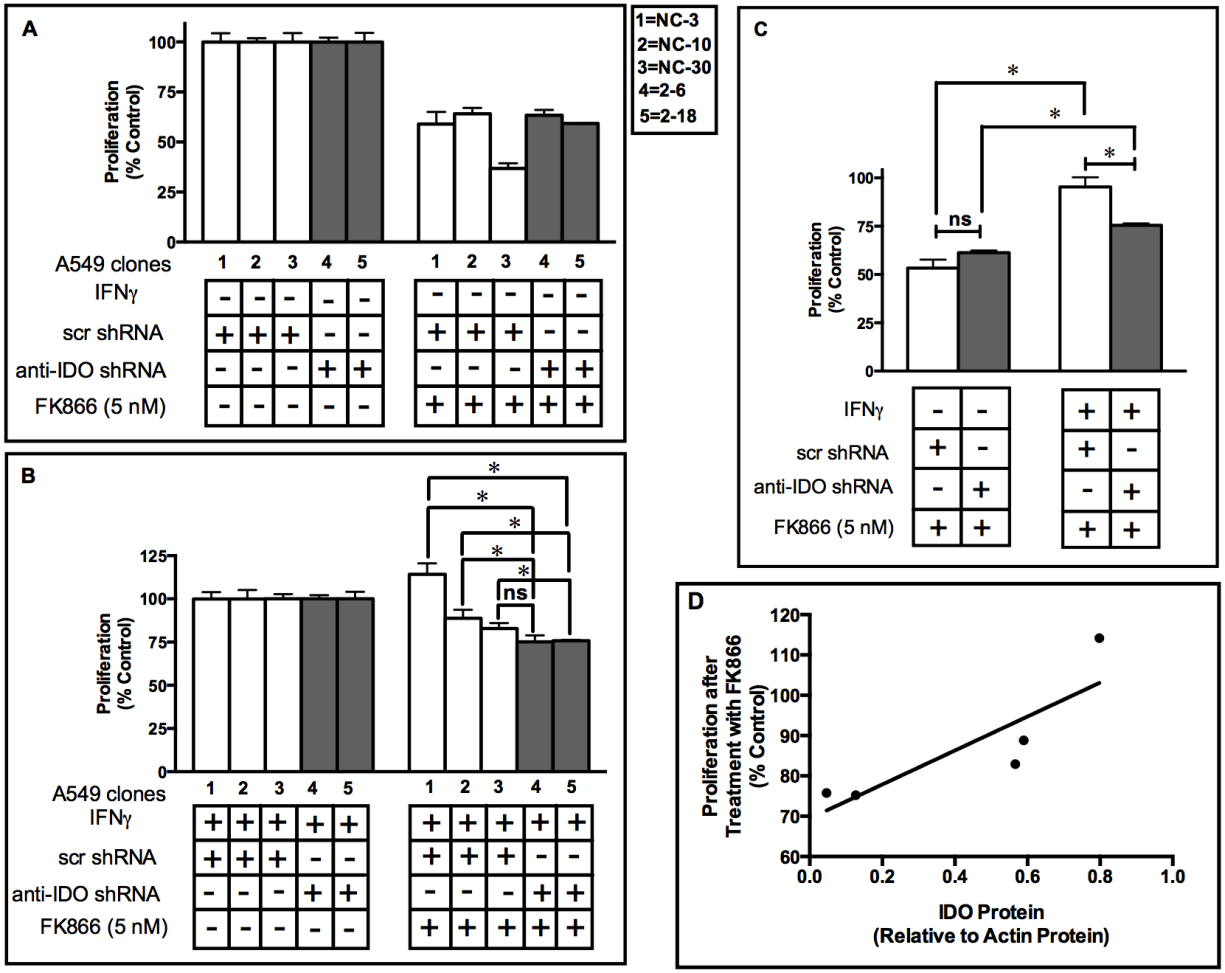
A549 clonal cell population sensitivity to FK866 (5 nM) before and after IDO induction. Proliferation of each of 5 individual A549 cell clonal populations before (**Panel A**) and after (**Panel B**) IDO induction with IFNγ. A549 clonal cell populations were cultured with or without IFNγ (25 ng/ml) for 48 h. Medium was then replaced with fresh growth medium containing FK866 (5 nM) and cells were allowed to proliferate for 72 h. Cells were then trypsinized and live cells were enumerated. **White bars**: A549 clones transfected with scrambled, non-targeting control shRNA. **Gray bars**: A549 cells transfected with anti-IDO shRNA. Results are normalized to control cells not treated with FK866, without (panel A) or with (panel B) IFNγ treatment. Each bar represents the mean of 3 values (*n* = 3) ± SD, (*, p≤0.05). **Panel C**: A549 clonal resistance to FK866 before and after IFNγ-mediated IDO induction. Pooled results were obtained from 3 or 2 independent A549 clonal populations stably transfected with scrambled shRNA (white bars) or 2 anti-IDO shRNA (black bars) respectively, before and after induction of IDO. Each bar represents a mean of 9 (white bars) or 6 (black bars) values ± SEM, Significant difference, Student's *t*-test, *p*<0.05. **Panel D**: Correlation analysis of the relationship between IDO protein content (relative to actin) and clonal population resistance to FK866 (proliferation relative to untreated control cells).

FK866 is a pharmacological inhibitor of NAD^+^ synthesis from the salvage pathway and is being evaluated for clinical anticancer efficacy [24]. IDO-expressing cancer cells have elevated NAD^+^ [4]. IDO downregulation also decreases intracellular NAD^+^ levels in cancer cells by 60% [4]. We therefore examined whether the IDO-mediated elevation in NAD^+^ levels has the potential to counter the therapeutic effect of FK866. After IFNγ stimulation of IDO, A549 clones NC-3, NC-10, and NC-30 (stably transfected with control, scrambled shRNA) showed greater resistance to the anti-proliferative effect of FK866 than A549 clones 2–6 and 2–8 (stably transfected with anti-IDO shRNA), with only the difference between clones NC-30 and 2–6 failing to achieve significance (Fig 1A and 1B). When data from the 3 clonal populations stably transfected with scrambled control shRNA were combined and compared to combined data from the 2 clonal populations stably transfected with anti-IDO shRNA, the former (*i*.*e*., A549 clones with unreduced IDO) were significantly more resistant (by approximately 20%) to FK866 after IFNγ induction of IDO (Fig 1C). There was no difference in sensitivity between clones harboring control scrambled shRNA and anti-IDO shRNA prior to IFNγ treatment, indicating that basal clonal characteristics, uninduced by IFNγ, were not responsible for differences in FK866 sensitivity. There was also a modest positive linear correlation (R^2^ = 0.72) between IDO protein levels after IFNγ induction measured in all 5 clonal populations, and resistance to FK866 (Fig 1D).

### IDO in Human Tumor Cells Mediates Resistance to the Base Excision Repair Inhibitor Methoxyamine

**Fig 2:**
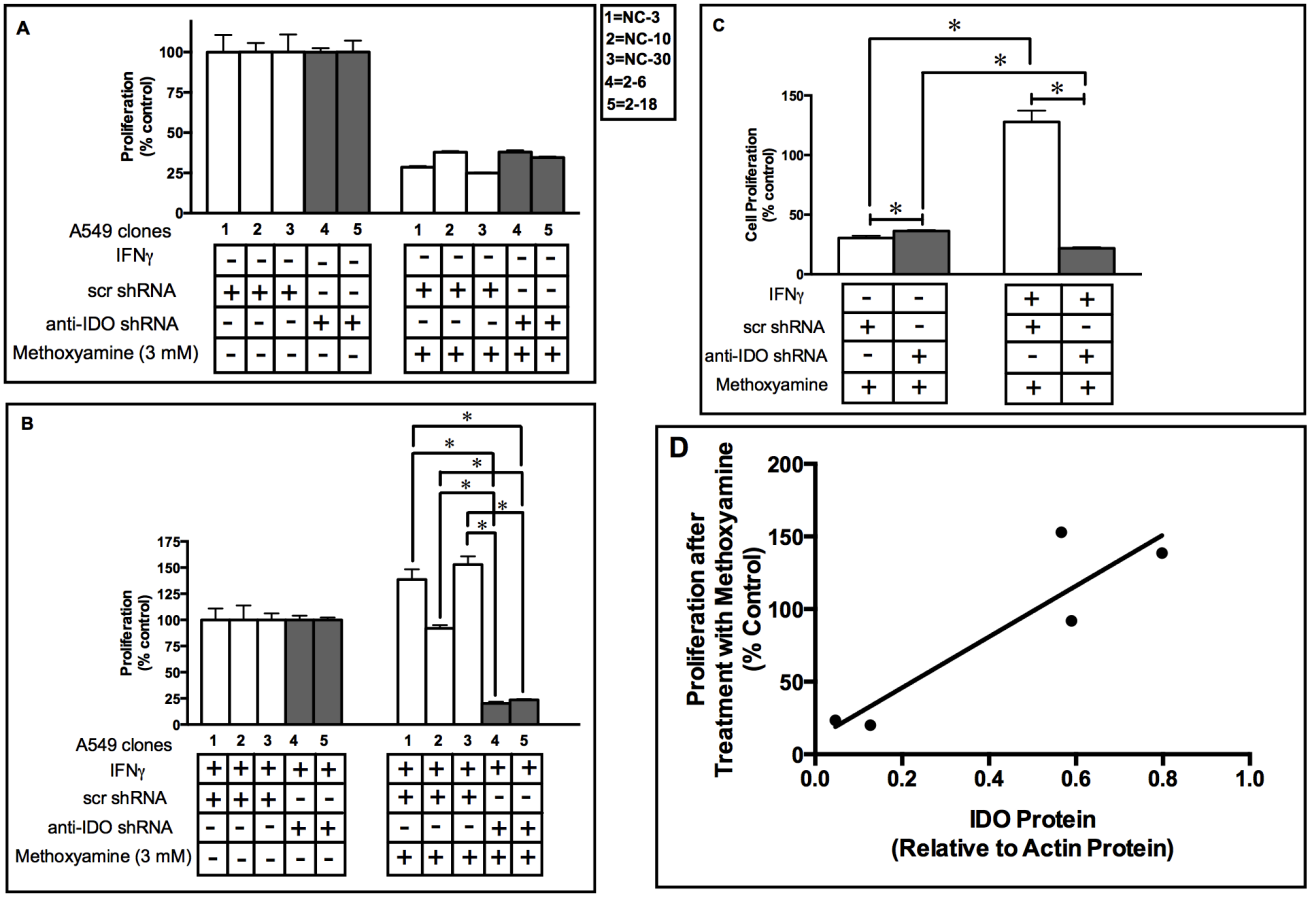
A549 clone sensitivity to methoxyamine (3 mM) before and after IDO induction. Proliferation of each of 5 individual A549 cell clonal populations before (**Panel A**) and after (**Panel B**) IDO induction with IFNγ. A549 clonal populations were cultured with or without IFNγ (25 ng/ml) for 48 h. Cultured medium was then replaced with fresh growth medium containing Methoxyamine (MX) (3 mM) and cells were allowed to proliferate for 72 h. Cells were then trypsinized and live cells were enumerated. **White bars**: A549 clones transfected with scrambled, non-targeting control shRNA. **Gray bars**: A549 cells transfected with anti-IDO shRNA. Each bar represents the mean of 3 values (*n* = 3 for determination of each value) ± SD. Results are normalized to control cells not treated with methoxyamine, without (panel A) or with (panel B) IFNγ treatment. **Panel C**: Induction of IDO in A549 clonal cell populations induces resistance to MX (3 mM). Results were obtained from 3 or 2 independent clonal cell populations with scrambled, non-targeting control shRNA or anti-IDO shRNA, respectively. Each bar represents a mean of 9 (white bars) or 6 (black bars) values ± SEM, *Significant difference, Student's *t*-test, *p*<0.05. **Panel D**: Relationship between IDO protein level (relative to actin) and resistance to methoxyamine (MX)(proliferation relative to untreated control cells). The R^2^ value of 0.83 represents a moderate positive relationship.

NAD^+^ is important for PARP function and PARP is essential for recruitment of the BER scaffold protein XRCC1 to damaged DNA [20]. In light of our previous report that IDO plays a role in mediating resistance to the PARP inhibitor olaparib [4], the capacity of IDO to mediate resistance to the BER inhibitor MX was examined. Antisense knockdown of IDO sensitized IFNγ-induced A549 clones 2–6 and 2–18 (harboring anti-IDO shRNA) to MX, while A549 clones NC-3, NC-10, and NC-30 (transfected with scrambled control shRNA) were not sensitized (Fig 2A and 2B). When data from the 3 clonal populations with control shRNA were combined and compared to combined data from the 2 clonal populations with anti-IDO shRNA, IFNγ-induced IDO mediated a 6-fold increase in resistance to the anti-proliferative effects of MX and the presence of anti-IDO shRNA abolished that increase (Fig 2C). There was a moderate correlation between IDO level and MX resistance among all 5 IFNγ-induced clonal populations [R^2^ = 0.83] (Fig 2D). These results suggest that IDO in cancer cells enhances the function of BER proteins, and antisense-mediated reduction of IDO blocks that enhancement.

### IDO in Human Tumor Cells Mediates Resistance to the TS-targeting Drug Pemetrexed

**Fig 3:**
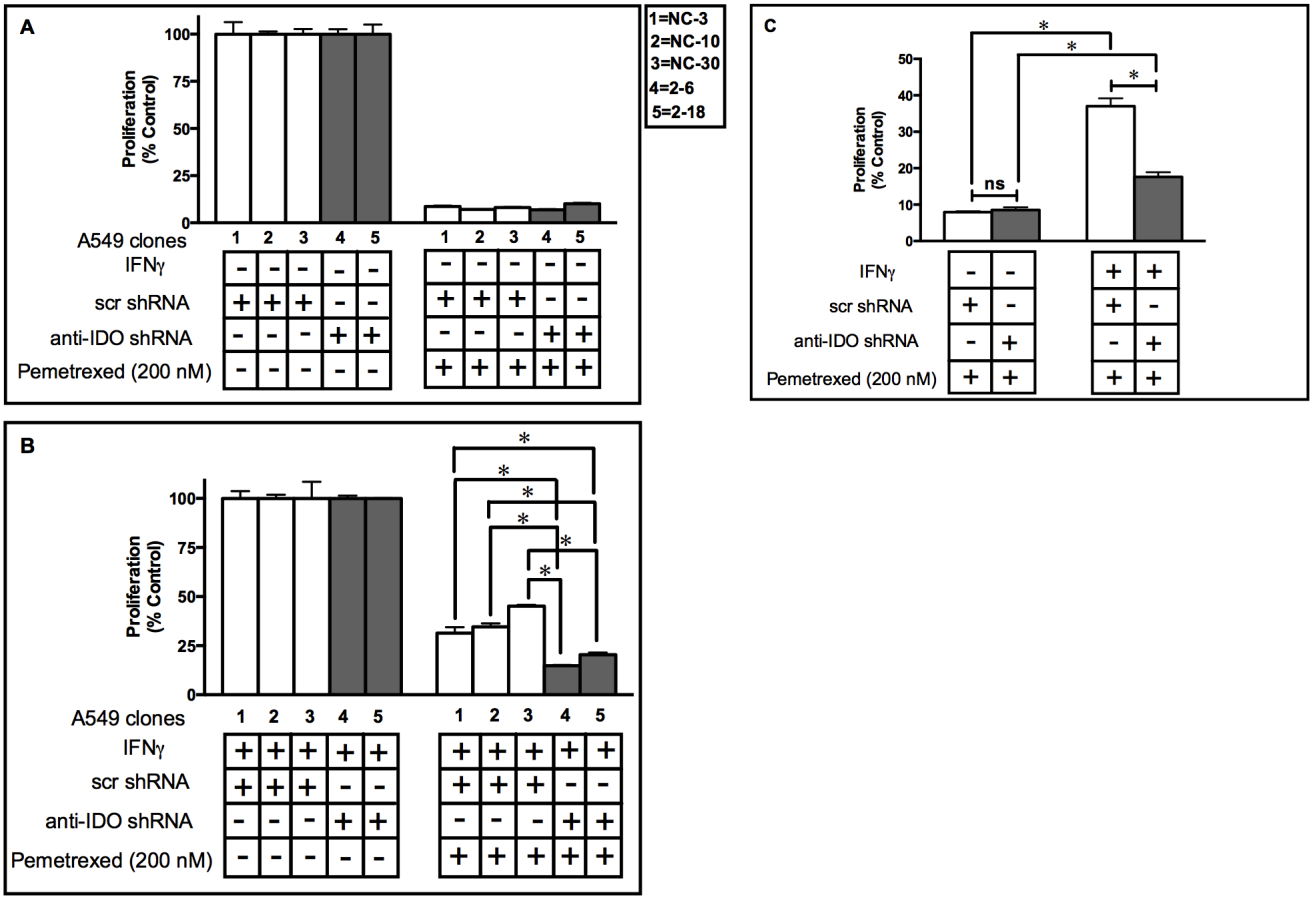
A549 clone sensitivity to pemetrexed (200 nM) before and after IDO induction. Proliferation of each of 5 individual A549 cell clonal populations before **(Panel A)** and after **(Panel B)** IDO induction with IFNγ. A549 clonal populations were cultured with or without IFNγ (25 ng/ml) for 48 h, then with pemetrexed (200 nM), and enumerated 72 h later. **White bars**: A549 clones transfected with scrambled, non-targeting control shRNA. **Gray bars**: A549 cells transfected with anti-IDO shRNA. Each bar represents the mean of 3 values (*n* = 3) ± SD. Results are normalized to control cells not treated with pemetrexed, without (panel A) or with (panel B) IFNγ treatment. **Panel C**: Induction of IDO in A549 clonal cell induces resistance to pemetrexed (200 nM). Results were obtained from 3 or 2 independent clonal cell populations with scrambled, non-targeting control shRNA or anti-IDO shRNA, respectively. Each bar represents a mean of 9 (white bars) or 6 (black bars) values ± SEM. *Significant difference, Student's *t*-test, *p*<0.05.

TS is important in DNA repair and DNA synthesis and is overexpressed in most human cancers [25]. The TS-targeting drug pemetrexed is commonly used to treat multiple types of human cancers including NSCLC and pleural mesothelioma [26]. BER is reported to be important in cancer cell resistance to this drug. We have shown before that IDO increased NAD^+^ levels in cancer cells [4]. In light of our findings that IDO conferred resistance to the BER inhibitor MX (Fig 2), we set to examined whether the presence of IDO affected the sensitivity of A549 clonal cell populations to pemetrexed, potentially linking IDO expression with enhanced BER function in cancer cells. Clonal A549 cell populations harboring scrambled control shRNA (3 clones) or anti-IDO shRNA (2 clones) were treated with or without IFNγ for 48 h and then with pemetrexed (200 nM) and allowed to proliferate for 72 h. There were no significant differences in sensitivity to pemetrexed among the 5 clones before IFNγ induction, whether the clones were assessed individually (Fig 3A) or mean relative sensitivity of the 3 control shRNA clones (n = 3 assessments of each clone) was compared to the mean relative sensitivity of the 2 anti-shRNA clones (n = 3 assessments of each clone)(Fig 3C). Antisense-mediated downregulation of IFNγ-induced IDO, in contrast, sensitized each of the 2 clones harboring anti-IDO shRNA to pemetrexed, compared to all 3 clones harboring scrambled control shRNA (Fig 3B). When mean proliferation of control and anti-IDO shRNA clones after IFNγ induction was compared, those harboring anti-IDO shRNA were approximately twice as sensitive to pemetrexed (Fig 3C). Antisense-mediated reduction in IDO, therefore, sensitized A549 clonal populations to pemetrexed.

### IDO in Human Tumor Cells Mediates Resistance to Combined Treatment of Pemetrexed and Methoxyamine

**Fig 4:**
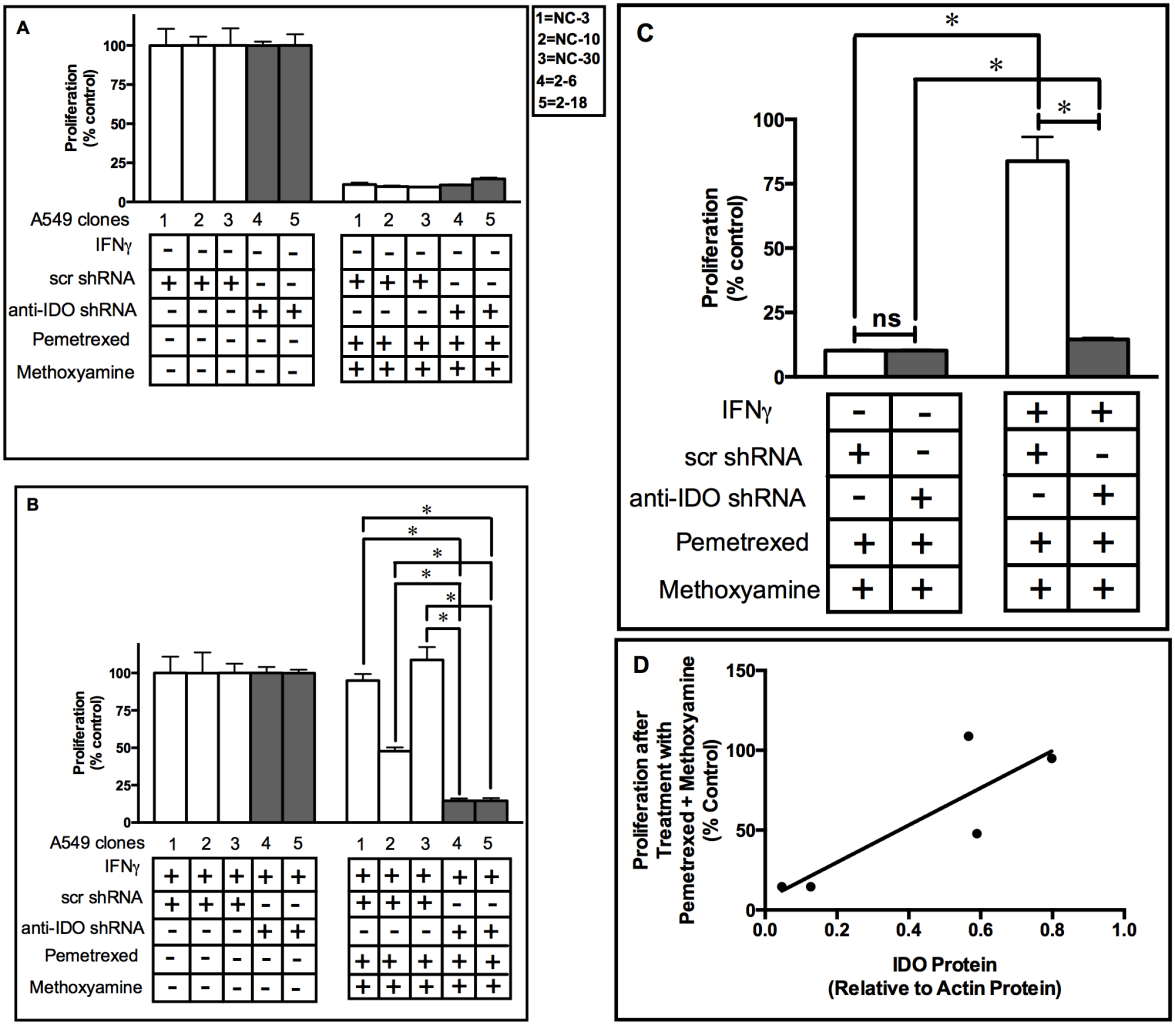
A549 clone sensitivity to combined pemetrexed and methoxyamine treatment before and after IDO induction. Proliferation of each of 5 individual A549 cell clonal populations before **(Panel A)** and after **(Panel B)** IDO induction with IFNγ. A549 clonal populations were cultured with or without IFNγ (25 ng/ml) for 48 h. Cultured medium was then replaced with fresh growth medium containing pemetrexed (30 nM) and methoxyamine (MX) (3 mM). Tumor cells were then allowed to proliferate for 72 h, then enumerated. **White bars**: A549 clones transfected with scrambled, non-targeting control shRNA. **Gray bars**: A549 cells transfected with anti-IDO shRNA. Each bar represents the mean of 3 values (*n* = 3) ± SD. *Significant difference, Student's *t*-test, *p*<0.05. Results are normalized to control cells not treated with pemetrexed and methoxyamine, without (panel A) or with (panel B) IFNγ treatment. **Panel C**: Induction of IDO in A549 clonal cell induces resistance to combined pemetrexed (30 nM) and MX (3 mM) treatment. Results were obtained from 3 independent clonal cell populations with scrambled control shRNA or anti-IDO shRNA, and each bar represents a mean of 9 (white bars) or 6 (black bars) values ± SEM (*p<0.05). **Panel D**: relationship between IDO protein (relative to actin) and clonal population resistance to combined pemetrexed and MX treatment proliferation relative to untreated control cells). The R^2^ value of 0.72 represents a moderate positive relationship.

A phase I clinical trial of combined MX and pemetrexed has been completed (NCT00692159) and phase II clinical trials of that drug combination in multiple indications including NSCLC are planned (NCT01851369, NCT02395692, NCT02535325, and NCT02535312) [26]. In view of our observation of IDO-mediated resistance to both pemetrexed and MX, it was hypothesized that IDO could induce resistance to combined MX and pemetrexed treatment. To test this hypothesis, IDO was induced in A549 clonal cell populations and then those populations were treated with a combination of pemetrexed (30 nM) and MX (3 mM) and proliferation assessed after 72 h. Similar to what was observed after treatment with MX or pemetrexed as single agents, IDO downregulation sensitized cancer cells to combined treatment (Fig 4A and 4B). Moreover, after IFNγ induction of IDO and exposure to combined pemetrexed and MX, when mean proliferation values for the 3 control clones were compared to mean proliferation values for the 2 control clones (n = 3 assessments for each clone), cells harboring anti-IDO shRNA were 7-fold more sensitive to the combined drug treatment than cells with scrambled control shRNA (Fig 4C). Prior to IFNγ induction of IDO expression, there was no difference (Fig 4C). There was a modest positive relationship between the amount of IDO in IFNγ-induced tumor cells and their resistance to combined treatment with these two drugs (Fig 4D, R^2^ = 0.70). These data indicate the significance of IDO in mediating resistance to therapeutic agents both alone and in combination.

### IDO Downregulation Sensitizes Human Tumor Cells to Gemcitabine

**Fig 5:**
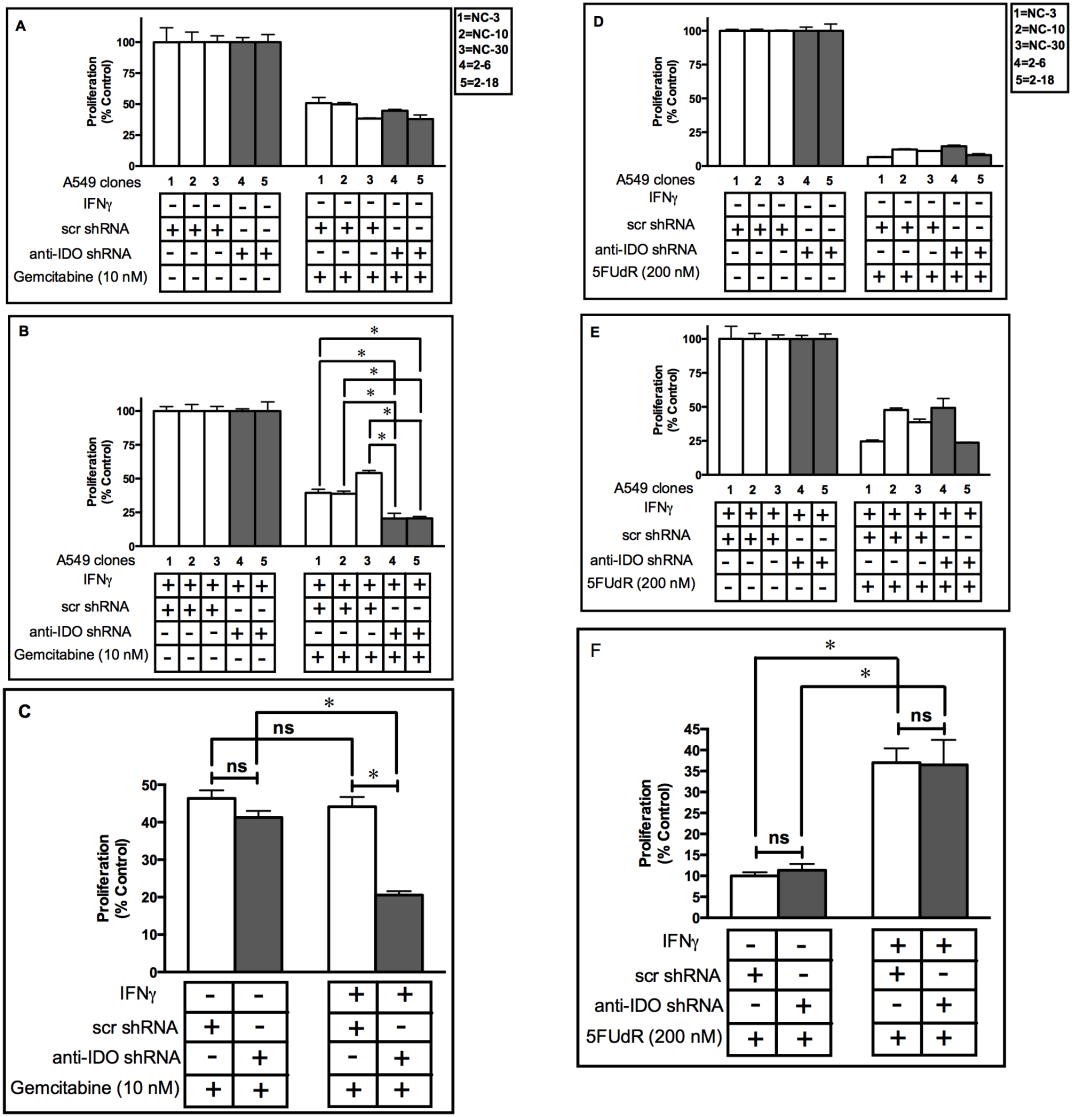
A549 clone sensitivity to Gemcitabine and 5FUdR before and after IDO induction. Proliferation of each of 5 individual A549 cell clonal populations before **(Panel A and C)** and after **(Panel B and C)** IDO induction with IFNγ. A549 clonal populations were cultured with or without IFNγ (25 ng/ml) for 48 h, treated with gemcitabine (10 nM) for 72 h, and then enumerated. **White bars**: A549 clones transfected with scrambled, non-targeting control shRNA. **Gray bars**: A549 cells transfected with anti-IDO shRNA. Each bar represents a mean of 9 (white bars) or 6 (black bars) values ± SD for Panels A and B and SEM for panel C, (*p<0.05). Results are normalized to control cells not treated with Gemcitabine, without (panel A) or with (panel B) IFNγ treatment. **Panels D-F**: Proliferation of each of 5 individual A549 cell clonal populations before and after IDO induction with IFNγ. A549 clonal populations were cultured with or without IFNγ (25 ng/ml) for 48 h and, 5FUdR (200 nM) for 72 h, and then enumerated. **White bars**: A549 clones transfected with scrambled, non-targeting control shRNA. **Gray bars**: A549 cells transfected with anti-IDO shRNA. Each bar represents a mean of 9 (white bars) or 6 (black bars) values ± SD for Panels D and E and SEM for panel F ± SD. *Significant difference, Student's *t*-test, *p*<0.05. Results are normalized to control cells not treated with 5FUdR, without (panel A) or with (panel B) IFNγ treatment.

IDO downregulation sensitized cancer cells to the TS-targeting drug pemetrexed (Fig 3). BER is also considered to play a major role in resistance to another TS-targeting drug, gemcitabine [27]. We therefore hypothesized that IDO downregulation would sensitize A549 cells to gemcitabine and, as described for pemetrexed and MX, this was the case (Fig 5A and 5B). When data from the 3 control clonal cell populations was pooled and compared to pooled data from the 2 anti-IDO-containing clonal populations (n = 3 assessments of proliferation for each clone), cells with anti-IDO shRNA were twice as sensitive to gemcitabine after IFNγ induction as control cells (Fig 5C). There was no difference between control and anti-IDO shRNA cells in sensitivity to gemcitabine before IFNγ treatment, nor was there a difference in sensitivity between control cells treated and untreated with IFNγ (Fig 5C). These data suggest that IDO downregulation can reduce BER-mediated treatment resistance in cancer cells.

### IDO Downregulation did Not Sensitize Human Tumor Cells to 5FUdR

In contrast to the role of BER in response to pemetrexed, MX, and gemcitabine, BER has been suggested to enhance rather than inhibit 5FUdR cytotoxicity in cancer cells, due to its participation in a futile repair cycle that potentiates 5FUdR toxicity [28]. To further determine whether BER in IDO-expressing clonal cells is involved in tumor cell resistance to pemetrexed and gemcitabine, we hypothesized that IDO downregulation would not increase 5FUdR effectiveness against A549 cells. In agreement to our hypothesis, IDO downregulation did not increase cancer cell sensitivity to 5FUdR (Fig 5D–5F). These data further support a role for IDO in modulation of BER in cancer cells.

### TS Downregulation Enhances the Capacity of IDO Downregulation to Sensitize A549 Cells to Pemetrexed

**Fig 6:**
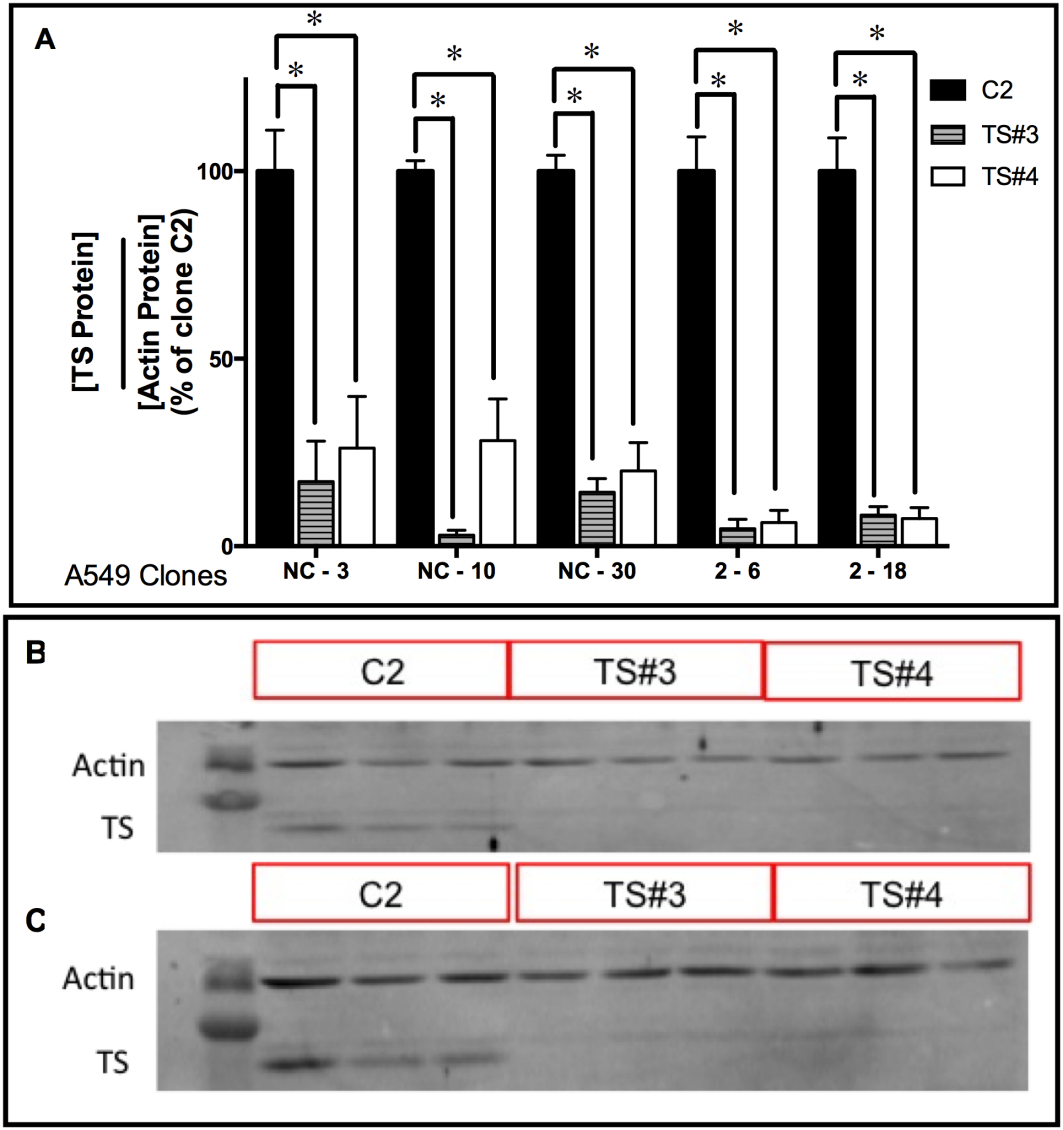
TS siRNA downregulation in A549 clonal populations. A549 clonal cell populations (NC-3, NC-10, and NC-30, each with stably-incorporated control, non-targeting scrambled shRNA; and 2–6 and 2–18, each with stably-incorporated anti-IDO shRNA) were seeded and grown overnight. Thymidylate synthase (TS) siRNA numbers 3 or 4, or scrambled, non-targeting control siRNA, were transiently transfected into each clonal population. Cells were lysed and protein was harvested 96 h later. TS and actin protein levels were determined by immunoblot. Results were quantified for each clone separately. **A**: TS protein quantification for each clonal population. Each bar represents the mean of 3 values (*n* = 3) ± SEM. *Significant difference from the same cells transfected with scrambled control, non-targeting siRNA, Student's *t*-test, *p*<0.05. **B**: Representative immunoblots of TS and actin protein in clone NC-3 after transfection of TS siRNA (siRNA numbers 3 and 4) or scrambled, non-targeting control siRNA (C2). **C**: Representative immunoblots of TS and actin protein in clone 2–18 after transfection of TS siRNA.

**Fig 7:**
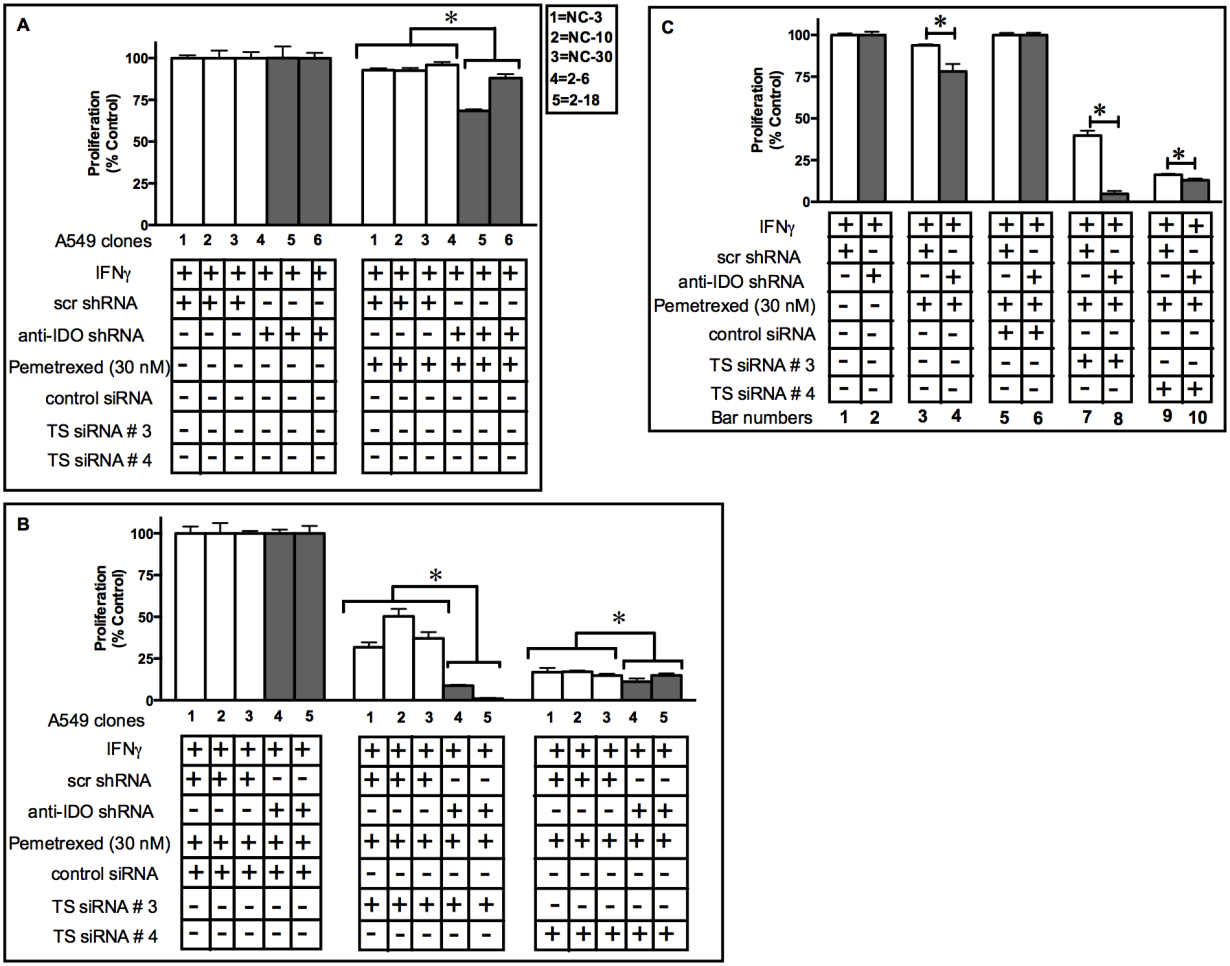
Concurrent IDO and TS downregulation sensitizes A549 cells to pemetrexed more than IDO knockdown alone. A549 cells were transfected with scrambled, non-targeting control siRNA or anti-thymidylate synthase (anti-TS) siRNA, treated with IFNγ (25 ng/ml) for 48 h, pemetrexed (30 nM) for 72, and then enumerated. Bars indicate mean relative cell numbers (n = 3 ± SD). **A**: Proliferation of clonal A549 cell populations induced with IFNγ and then treated with pemetrexed, but untransfected with siRNA of any kind. **Gray bars**: clones containing anti-IDO shRNA. **White bars**: clones containing scrambled, non-targeting control shRNA. **B**: Proliferation of the same clonal A549 cell populations transfected with scrambled, non-targeting control siRNA, TS siRNA #3, or TS siRNA #4, induced with IFNγ, and then treated with pemetrexed. Bars represent values normalized to values obtained from clones treated with IFNγ but untreated with pemetrexed or siRNA; those cells were each considered to have a proliferation value of 100% after IFNγ treatment. **Gray bars**: clones containing anti-IDO shRNA. **White bars**: clones containing non-targeting control shRNA. *Significant difference, Student's *t*-test, *p*<0.05. Data presented a representative experiment from two independent experiments. Results are normalized to control cells not treated with pemetrexed, but with treated with IFNγ. **Panel C**: shows the pooled results from panel A and B.

TS mRNA downregulation sensitizes A549 cells to the TS-targeting drug 5FUdR [29]. IDO downregulation sensitized A549 cells to the TS-targeting drugs pemetrexed and gemcitabine (Figs 3 and 5) but not 5FUdR (Fig 5). To test whether concurrent knockdown of both TS and IDO sensitized A549 cells to anti-TS drugs more effectively than knockdown of IDO alone, A549 clonal populations (stably transfected with anti-IDO shRNA or control shRNA) were transiently transfected with two TS siRNAs (Table 1). Two different TS siRNAs downregulated TS protein by 70–99% in all five A549 clonal populations (harboring either control non-targeting shRNA or anti-IDO shRNA) by 96 h post-transfection (Fig 6A and 6B). There was no difference in the degree of antisense-mediated reduction in TS when cells induced with IFNγ were compared to uninduced cells, nor did transfection of anti-TS siRNA alter IFNγ induction of IDO (data not shown). Concurrent IDO and TS downregulation sensitized cancer cells to pemetrexed more effectively than knockdown of IDO alone (Fig 7A and 7C): when pooled data from the 3 control clones and 2 anti-IDO shRNA clones were compared, knockdown of IDO alone increased sensitivity to pemetrexed by approximately 17% (Fig 7C, bar 3 vs bar 4) and knockdown of TS alone (TS siRNA #3) by 60% (Fig 7C, bar 5 vs bar 7), but knockdown of both IDO and TS (TS siRNA #3) increased sensitivity by 95% (Fig 7C, bar 8). Combined knockdown of IDO and TS using a different anti-TS siRNA (TS siRNA #4) was also significantly more effective than either knockdown alone, but to a lesser degree (Fig 7C, bar 10).

### IDO Downregulation Enhances the Capacity of TS Downregulation to Sensitize A549 Cells to 5FUdR

**Fig 8:**
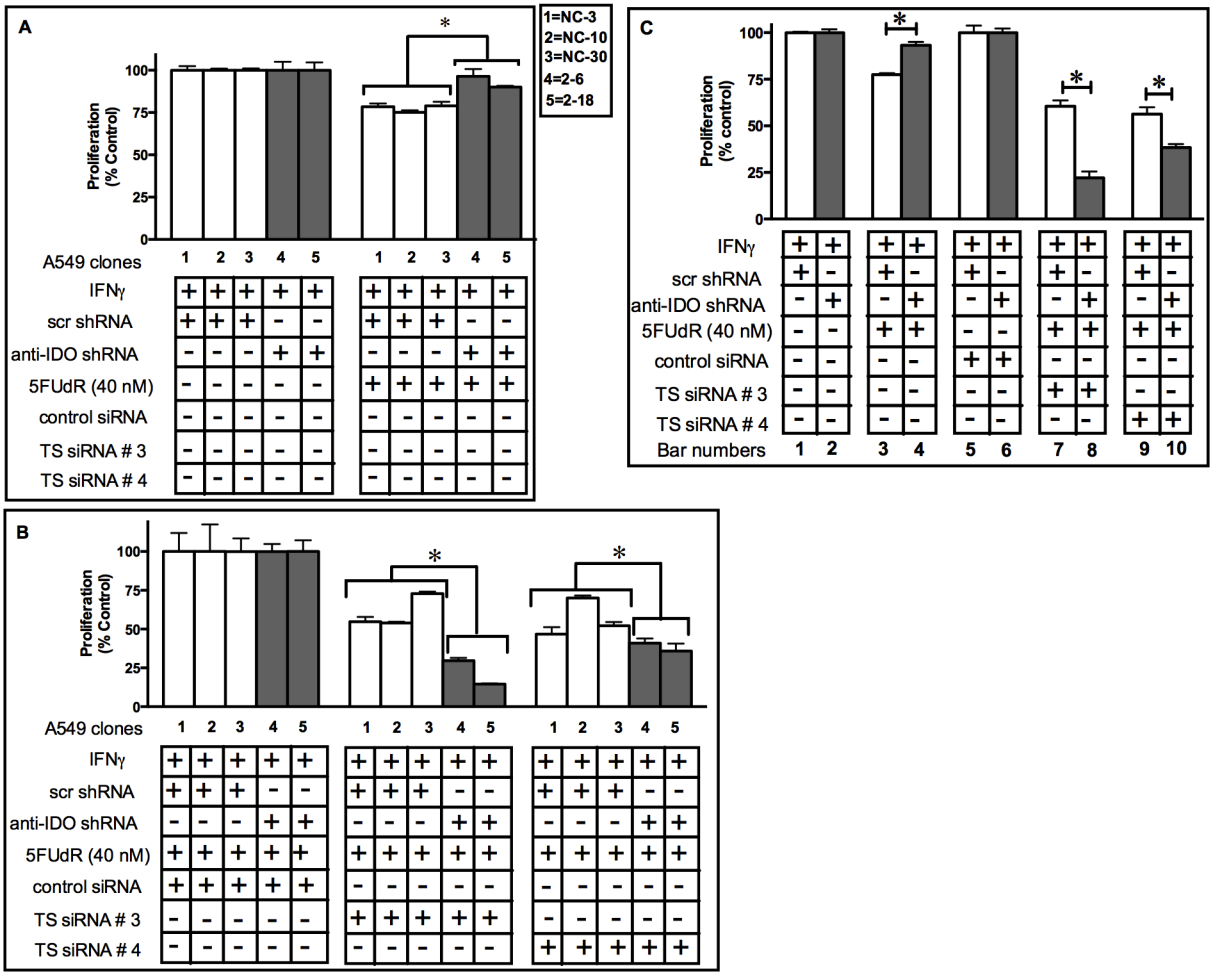
Concurrent IDO and TS downregulation sensitizes A549 cells to 5FUdR more than TS knockdown alone. A549 cells were transfected with control or anti-thymidylate synthase (anti-TS) siRNA, treated with IFNγ (25 ng/ml) for 48 h, with 5FUdR (40 nM) for 72 h, and then enumerated. Bars indicate mean proliferation relative to appropriate controls ± SD (n = 3). **A**: Proliferation of clonal A549 cell populations induced with IFNγ and then treated with 5FUdR, but untransfected with siRNA of any kind. **Gray bars**: clones containing anti-IDO shRNA. **White bars**: clones containing non-targeting control shRNA. **B**: Proliferation of the same clonal A549 cell populations transfected with control non-targeting siRNA, TS siRNA #3, or TS siRNA #4, induced with IFNγ, and then treated with 5FUdR. Bars represent values normalized to values obtained from clones treated with IFNγ but untreated with pemetrexed or siRNA; those cells were considered to have a proliferation value of 100% after IFNγ treatment. **Gray bars**: clones containing anti-IDO shRNA. **White bars**: clones containing non-targeting control shRNA. *Significant difference, Student's *t*-test, *p*<0.05. Data presented a representative experiment from two independent experiments. Results are normalized to control cells not treated with 5FUdR, but with treated with IFNγ. **Panel C**: shows the pooled results from panel A and B.

Combined antisense downregulation of IDO and TS sensitized A549 cells to the TS-targeting drug pemetrexed to a greater degree than antisense downregulation of TS alone (Fig 7). In addition, IDO downregulation alone did not enhance A549 cell sensitivity to 5FUdR (Fig 5). We therefore assessed the capacity of combined, concurrent downregulation of both IDO and TS downregulation to sensitize human tumor cells to 5FUdR to a greater degree than TS downregulation alone. Concurrent IDO and TS downregulation using TS siRNAs numbers 3 or 4, combined with shRNA-mediated reduction of IDO in response to induction with IFNγ, sensitized cancer cells to 5FUdR to a greater degree than TS downregulation alone. Knockdown of IDO alone did not increase sensitivity to 5FUdR and, in fact, increased 5FUdR resistance to a small degree (by approximately 20%)(Fig 8C, bar 3 vs. bar 4), in agreement data shown in Fig 5. Knockdown of both IDO and TS (TS siRNA #3) enhanced sensitivity by 65% compared to knockdown of TS alone (Fig 8C, bar 7 vs. bar 8). When a different TS siRNA (TS siRNA #4) was used in combination with knockdown of IDO, sensitivity was enhanced by 30% (Fig 8C, bar 9 vs. bar 10).

### Concurrent IDO and BRCA2 Downregulation Does Not Sensitize A549 Cells to 5FUdR

**Fig 9:**
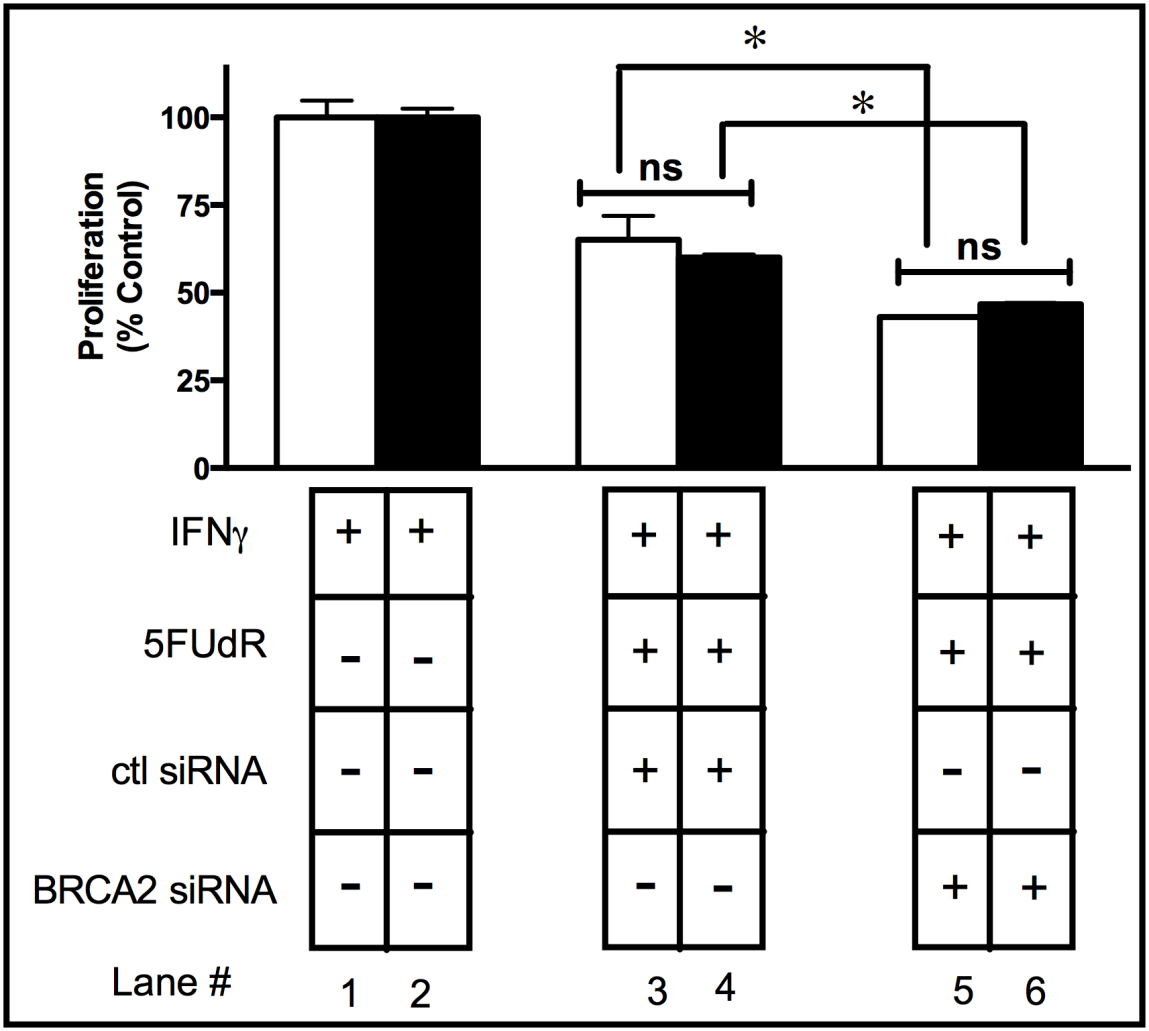
Concurrent downregulation of IDO and BRCA2 did not sensitize A549 to 5FUdR. A549 clonal cells transfected with either scrambled shRNA (NC-3) or anti-IDO shRNA (2–18) were transiently transfected with breast cancer type-2 susceptibility protein (BRCA2) siRNA, induced with IFNγ (25 ng/ml) for 24 h, and then treated with 5FUdR (40 nM) for 72 h, at which time live cells were enumerated. Simultaneous downregulation of IDO and BRCA2 did not sensitize A549 to the TS-targeting drug 5FUdR to a greater degree than the knockdown of either gene alone. Bars represent the means of 3 independent measurements of cells (with or without downregulation of IDO) after BRCA2 siRNA transfection + 5FUdR treatment (*n* = 3 for each measurement) ± SD. Bars were normalized to values obtained from clones treated with IFNγ but untreated with 5FUdR or siRNA; those cells were considered to proliferate at a 100% level after IFNγ treatment. *Significant difference, Student's *t*-test, *p*<0.05.

Antisense knockdown of IDO enhanced the capacity of antisense knockdown of TS to sensitize human tumor cells to 5FUdR (Fig 8). To further establish the specific role of IDO on BER and not other DNA repair pathways, the capacity of antisense knockdown of IDO combined with BRCA2 knockdown to sensitize human tumor cells to 5FUdR was evaluated (Table 1). BRCA2 is vital for homologous recombination repair and is not involved in BER [30]. Antisense reduction of IDO alone did not sensitize A549 cells to 5FUdR (Fig 9, lane 3 vs. lane 4), but antisense downregulation of BRCA2 sensitized A549 cells to 5FUdR (Fig 9, lane 3 vs. lane 5). Notably, concurrent downregulation of IDO and BRCA2 did not sensitize cancer cells to 5FUdR to any greater degree than knockdown of BRCA2 alone (Fig 9, lane 5 vs. lane 6). These results suggest that knockdown of IDO does not contribute to sensitization to the TS-targeting drug 5FUdR, either alone or in combination with knockdown of BRCA2 that operates outside BER.

### IDO Downregulation Sensitizes A549 Tumor Xenografts Treated with Pemetrexed

**Fig 10:**
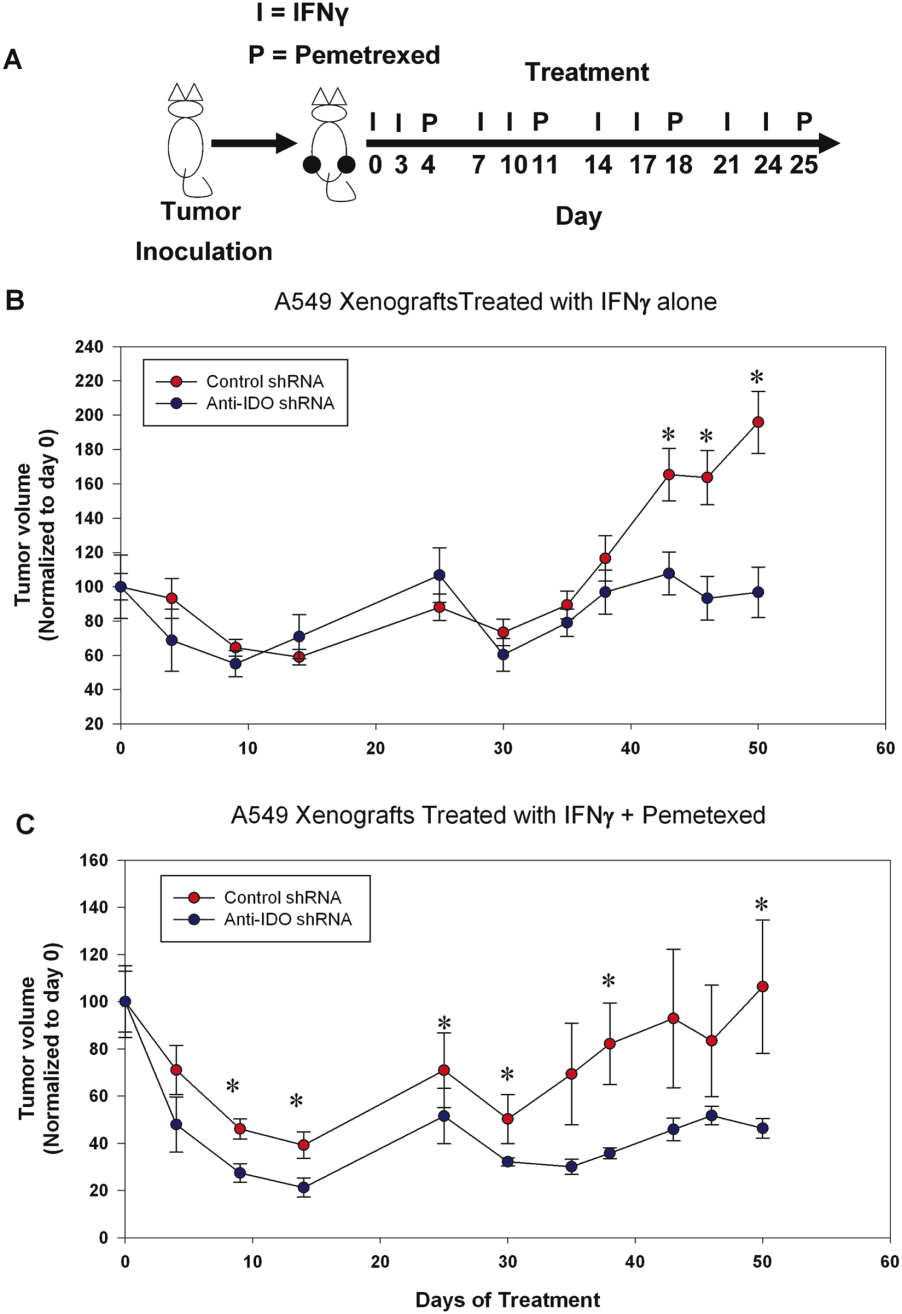
IDO downregulation sensitized A549 xenografts to pemetrexed treatment. **A**: Ten million A549 cells transfected with control shRNA (clone NC-3) or anti-IDO shRNA (clone 2–18) were injected into flanks of SCID mice. Once tumors reached ~300 mm^3^ animals were injected with 100,000 IU IFNγ i.p. (twice a week for four weeks) and 50 mg/kg pemetrexed i.p. (once per week for four weeks). Animals transplanted with A549 clonal cells transfected with control shRNA or anti-IDO shRNA received four weeks of IFNγ treatment alone **(B)** or IFNγ and pemetrexed **(C)**. Tumor growth was monitored with caliper measurement to estimate tumor volume. *Significant difference, Student's *t*-test, *p*<0.05.

IDO downregulation sensitized A549 clonal populations to pemetrexed *in vitro* (Fig 3). We sought to examine the effect of IDO downregulation in an immunocompromised mouse model lacking B and T cells. We implanted A549 clonal cell populations (NC-3 and 2–18) into the flanks of SCID mice and allowed xenografts to grow to ~300 mm^3^ before starting treatments with pemetrexed (Fig 10A). A549 cells do not naturally express IDO. We therefore treated the mice with IFNγ twice a week for four weeks to induce IDO expression in the xenografts. Animals did not show any side effects or weight loss during the course of treatment with IFNγ and pemetrexed (data not shown). Notably, A549 clones NC-3 and 2–18 showed similar proliferation rates before IDO induction *in vitro* (Fig 3A) and *in vivo* (data not shown). Interestingly, A549 xenografts transfected with anti-IDO shRNA showed significant delay in tumor growth in the presence of IFNγ alone when compared to A549 xenografts capable of producing IDO in the presence of IFNγ (Fig 10B). Most importantly, A549 xenografts transfected with anti-IDO shRNA were more sensitive to pemetrexed treatment compared to xenografts transfected with control shRNA (Fig 10C).

## Discussion

Most human tumors express IDO *in vivo* [7] and that expression plays a role in protecting tumors from identification and destruction by the immune system [7]. IDO-mediated resistance to ipilimumab [14] and endogenous anti-tumor immune activity [16] have sparked interest in development of new IDO inhibitors for potential combination with other immunotherapy drugs in treatment of cancer (NCT01792050). We have reported that IDO confers resistance to the PARP inhibitor olaparib, ionizing radiation, and cisplatin, independent of its immune function [4]. IDO expression also increases intracellular NAD^+^ levels required for PARP and BER activity [31]. IDO downregulation decreased intracellular NAD^+^ levels in A549 cells by approximately 60% [4]. FK866 is a potent NAD^+^ inhibitor that blocks NAD^+^ production through the salvage pathway [32] and is currently under clinical investigation [33]. Here we show, for the first time, that IDO confers resistance to the NAD^+^ inhibitor FK866 (Fig 1). This is significant with respect to the capacity of FK866 to block NAD^+^ synthesis in the presence of IDO. Because IDO-mediated NAD^+^ production from the *de novo* pathway can clearly undermine FK866 efficiency (Fig 1). In addition, tumor-infiltrating cytotoxic T cells and NK cells are major sources of IFNγ in the tumor microenvironment thus increasing IDO levels [34,35] and IFNγ-mediated increase in IDO-induced resistance to FK866 (Fig 1). Therefore, blocking IDO in conjunction with FK866 treatment may have therapeutic value and further studies are required.

The potential role of IDO in BER is unknown. IDO induced high levels of resistance to MX in A549 cells and that resistance was abolished by anti-IDO shRNA (Fig 2). Higher IDO levels were also positively correlated to MX resistance in cancer cells (Fig 2). These data further support the hypothesis that BER activity may be increased in human tumor cells due to an IDO-mediated increase in NAD^+^ levels. Several phase I clinical trials of combined MX with chemotherapy drugs are currently under way (NCT00892385, NCT00692159, and NCT02535312) [36]. One clinical trial in particular has studied the combined effect of MX and the TS-targeting drug pemetrexed in patients with advanced refractory cancers [36]. Therefore, consideration of potent IDO-mediated induction of resistance to MX could be critical in designing pre-clinical and clinical studies in future.

Pemetrexed inhibition of TS results in misincorporation of uracil into DNA. The BER enzyme uracil-DNA glycosylase (UNG) removes the misincorporated uracil and, by mediating that process, confers resistance to pemetrexed [37]. IDO inhibited the effectiveness of BER inhibitor MX (Fig 2). We therefore assessed whether IDO downregulation sensitized cancer cells to pemetrexed. Antisense knockdown of IDO did, in fact, sensitize A549 cells to pemetrexed both *in vitro* and *in vivo* (Figs 3 and 10C). Here we show, for the first time, that in response to a DNA-damaging chemotherapy drug, pemetrexed, and in the absence of T cells, IDO-downregulated xenografts are more sensitive to treatment. In addition, IDO-mediated resistance to pemetrexed was decreased by anti-IDO shRNA after IFNγ induction of IDO in A549 cells (Fig 3C). Since IFNγ has been shown to induce only the enzymatic function of IDO and not its signaling function [38], it is most likely that the role of IDO in inducing resistance to pemetrexed in our system is through its enzymatic function.

Interestingly, A549 xenografts stably transfected with control shRNA showed increased tumor growth when treated with IFNγ alone compared to the A549 xenografts transfected with anti-IDO shRNA (Fig 10B). Since the Fox Chase SCID mice used lack T and B cells, but have intact macrophage and NK cells, these results are in agreement with a previous finding that showed IDO overexpression induced rapid tumor growth in nude mice having intact NK populations [39].

Pemetrexed-resistant sublines of H1299 adenocarcinoma cells have elevated levels of UNG, hence enhanced BER activity, and combined treatment of these H1299 sublines with MX and pemetrexed increased their sensitivity to pemetrexed [37]. We therefore tested whether IDO in tumor cells can mediate resistance to combined pemetrexed and MX treatment. IFNγ-induced IDO undermined the therapeutic potential of the combined treatment of pemetrexed and MX (Fig 4). This effect was significantly reduced by anti-IDO shRNA in A549 clonal sublines (Fig 4). Moreover, IDO levels were positively correlated with resistance to combined pemetrexed and MX treatment in A549 clonal cells (Fig 4). These results provide compelling evidence for a previously unidentified role for IDO in induced resistance to a combination of the TS-targeting drug pemetrexed and the BER inhibitor MX. IDO is expressed in most late-stage human cancers [7]. Our findings underlie its critical role in inducing resistance to single- and multi-agent chemotherapy regimens currently under development, independent of its immune activity.

BER is considered to play a major role in resistance to another TS-targeting agent gemcitabine [27,40]. Since IDO downregulation sensitized cancer cells to pemetrexed, we hypothesized IDO may also be involved in BER-mediated gemcitabine resistance in these cells. Our data show that IDO downregulation sensitized cancer cells to gemcitabine. To further examine the role of IDO-mediated BER in the sensitivity of cancer cells to these TS-targeting agents, we decided to determine whether IDO downregulation sensitizes A549 cells to 5FUdR. The TS-targeting drug 5FUdR requires BER, to some extent, to exert its effect on cancer cells, contrary to the requirements of pemetrexed and gemcitabine [41,42]. Therefore, decreasing IDO-mediated BER by antisense knockdown of IDO was predicted to not increase the effectiveness of 5FUdR in A549 cells. In fact IDO downregulation did not sensitize cancer cells to 5FUdR (Fig 5). These data shed light, for the first time, on a previously unidentified mechanism of IDO function in DNA repair in human cancer cells.

Simultaneous downregulation of IDO and TS also increased the sensitivity of cancer cells to pemetrexed and 5FUdR to a greater degree than reduction of either target alone. TS siRNA downregulation has been shown to sensitize A549 cells to pemetrexed [43]. We showed here that combined TS and IDO downregulation further sensitized A549 cells to both pemetrexed (Fig 7) and 5FUdR (Fig 8). The additive effect of TS and IDO downregulation in A549 cells sensitivity to pemetrexed may be a result of the negative effect of reduced TS on the amount of available thymidylate in cells [44] along with the IDO-mediated impact on BER (Fig 2). These observations could provide the basis for a strategy to improve the effectiveness of the already approved chemotherapy drugs pemetrexed and 5FUdR.

To further examine the role of IDO on DNA repair pathways and whether concurrent IDO and TS downregulation have value in sensitizing human tumor cells to the TS-targeting drug 5FUdR, we simultaneously downregulated IDO and BRCA2 (a DNA repair molecule not involved in enzymatic reactions mediated by TS), in A549 cells followed by treatment with 5FUdR. BRCA2 does not mediate BER [45] and, therefore, it is unlikely that, by targeting BRCA2 (which involves DNA repair pathways other than BER), cancer cells would be sensitized to a drug that requires BER for its toxicity. As shown in Fig 9, combining IDO and BRCA2 downregulation did not sensitize cancer cells to 5FUdR. These data emphasize the importance of simultaneous knockdown of IDO, and a DNA repair molecule, to sensitize cancer cells to a drug that requires that specific DNA repair molecule for survival. In other words, reduction of IDO and BRCA2 does not appear to sensitize cancer cells to a drug such as 5FUdR that targets TS.

In summary, we identified IDO-mediated BER to be important in the resistance of human cancer cells to the widely-used chemotherapy drugs pemetrexed and gemcitabine, and agents currently under clinical investigation (FK866 and MX).

## Supporting Information

S1 Table Contains Minimal Data Set for these and all other experiments. (XLSX)
